# High efficient and cost-effective screening method for diabetic cardiovascular risk

**DOI:** 10.1186/1758-5996-6-51

**Published:** 2014-04-08

**Authors:** Tadafumi Kajimoto, Mami S Sawamura, Reiko D Hayashi, Takeshi Oya, Rieko A Hirao, Haruhiko Kouhara

**Affiliations:** 1 Department of Internal Medicine of Endocrinology and Metabolism, National Hospital Organization, Osaka Minami Medical Center, 2-1 Kidohigashimachi, Kawachinagano, Osaka 586-8521, Japan

## Abstract

**Background:**

The vascular complications of outpatients with diabetes at ordinary hospitals vary. Ischemic heart disease is barely predictable after treatment using previously reported therapeutic indices. We developed a simple and noninvasive screening method to evaluate the possibility of ischemic heart disease in patients with diabetes.

**Methods:**

Five years of clinical data from 337 outpatients (196 males and 141 females) with diabetes were analyzed. Twenty-three males and 14 females had ischemic heart disease. We examined the possibility of predicting ischemic heart disease after analyzing this population. The analyzed laboratory data included the following: minimum value of right or left ankle–brachial indices (ABI), maximum value of right or left pulse wave velocities (PWV), aortic calcification diagnosed on plain chest radiographs, plaque score (PS), maximum value of intima media thickness at the cervical artery (IMT), electrocardiographic (ECG) ischemic changes (including ST-T changes or abnormal Q waves, which were re-examined by a cardiologist), HbA1c, low-density lipoprotein cholesterol (LDL-C), uric acid (UA), urine albumin, age, sex, disease duration, and body mass index. All data were subjected to multivariate logistic regression analyses.

**Results:**

The presence of ECG ischemic changes, aortic calcification, minimum ABI, maximum IMT, LDL-C, and UA were evaluated in multivariate logistic regression analysis with the onset of ischemic heart disease. The receiver operating characteristic curve indicated an area under the curve of 0.879 (0.820 - 0.938; P = 0.00).

**Conclusions:**

Ischemic heart disease could be predicted in patients with diabetes using a combination of results from conventional physical and laboratory tests.

## Introduction

The evaluation of diabetic microangiopathies is well established, and there are many ways to predict prognoses. Although ischemic heart disease is lethal, the prognosis of diabetic macroangiopathy cannot be diagnosed without invasive procedures and computed tomography of the coronary arteries.

Contrast material may induce acute renal failure when a patient with diabetes is subjected to a radiological procedure [1]. Moreover, a large population cannot be surveyed with invasive tests because of the high cost. Therefore, a prescreening test is mandatory before invasive procedures. Useful procedures to predict ischemic risk have been reported, such as the Framingham score, maximum intima media thickness (IMT), ankle–brachial index (ABI), pulse wave velocity (PWV), aortic calcification, and coronary artery calcium score [2-6].

However, the Framingham score does not reflect the ongoing state of atherosclerosis; it only evaluates future risks caused by the metabolic condition attributed to a patient’s lifestyle. The Framingham study may have underestimated risk because it analyzed a large untreated population and, consequently, could not evaluate outpatients with diabetes and atherosclerosis. Generally, atherosclerosis has already progressed in patients with glucose intolerance, and atherosclerotic risk increases continuously after a diagnosis of diabetes is made. Our population with diabetes was different from that in the Framingham study [7,8]. Interestingly, situations like this in diabetes mellitus have also been found in chronic inflammatory rheumatic diseases such as rheumatoid arthritis; a disease associated with accelerated atherosclerosis with an increased risk of cardiovascular death similar to diabetes mellitus [9]. In rheumatoid arthritis, risk charts such as the Systematic COronary Risk Evaluation (SCORE) used to establish 10-year risk of fatal cardiovascular event, underestimate the actual cardiovascular risk of these patients. In this regard, recent studies have disclosed that patients with rheumatoid arthritis included in the category of moderate risk according to the SCORE risk chart have severe subclinical atherosclerosis when carotid ultrasound is performed [10,11].

An evaluation of complication risk is practically difficult because each outpatient is at a different stage of atherosclerosis during prognosis prediction. Moreover, predicting cardiovascular risk from the Framingham score is superfluous after treatment initiation.

Some studies have indicated that prognosis can be predicted by a single physical test such as measurement of maximum IMT, ABI, or PWV. Other studies have suggested that several laboratory tests can be combined and scored to predict the atherosclerotic prognosis in patients with diabetes.

Most of these studies analyzed a general population using radiation equipment during a mass screening; however, these tests have irradiation-associated side effects [12-14]. To overcome these problems, we studied an alternative method to combine several conventional tests and patient data to score the risk of ischemic heart diseases in patients with diabetes.

## Methods

A total of 337 patients (196 males and 141 females) with diabetes who visited our hospital between June 2008 and June 2013 were retrospectively analyzed. We performed physiological and imaging tests, including ABI measurement, plain chest radiography, cervical ultrasonography, annual electrocardiography, and blood tests. The lattermost included uric acid (UA) and the measurement of HbA1c, low-density lipoprotein cholesterol (LDL-C), and urinary albumin.

These tests were selected because they relate to ischemic heart disease and are used in routine clinical practice [15].

Our study was approved by the ethics committee of our hospital. The patients’ clinical data were retrieved from our hospital host computer.

We selected the latest ABI measurement, plain chest radiograph, and cervical ultrasonography data that reflected the progression of atherosclerosis. Abnormal ECGs were re-examined by a cardiologist. We regarded the test as positive if ST-T changes or abnormal Q waves appeared once in 5 years. However, in patients who developed ischemic heart disease in the past five years, we did not consider both the ECG findings after treatment and ECG findings in cardiac attack, but the ECG before the ischemic attack.

Medications such as hypoglycemic agents, statins, and anti-hyperuricemic drugs were not considered. We used the mean values of the laboratory data for 5 years because short-term variations in each value can mask the long-term metabolic state.

Disease duration and body mass index (BMI) data from 2008 were selected for analysis. Carotid artery ultrasonography and ABI included two values for the left and right sides. The larger value that reflected the progression of atherosclerosis was selected for analysis.

A smaller ABI value indicates a higher risk of ischemic heart disease. We selected either the right or left ABI that was smaller (minimum ABI value). We also chose each test that is conventionally used and has some relationship with atherosclerosis [2-6]. Among the 337 patients (196 males and 141 females) with diabetes, 37 patients (23 males and 14 females) suffered an ischemic heart disease within 5 years. Diagnosis of ischemic heart disease defined as the endpoint was performed with cardiac catheterization. Ischemic heart disease included 27 stable angina, 3 unstable angina, 4 non ST elevation myocardial infarction (NSTEMI), and 3 ST elevation myocardial infarction (STEMI).

## Results

The clinical characteristics of the 337 patients are shown in Table 1. Their mean age was 68.3 ± 10.2 years and their mean BMI was 24.1 ± 4.1 kg/m^2^. The mean disease duration was 14.2 ± 9.7 years. Seventy-eight patients exhibited ST-T changes and abnormal Q waves on their electrocardiogram (ECG), while 135 exhibited aortic calcification on a plain chest radiograph. The minimum ABI value was 1.08 ± 0.13, maximum PWV was 1830 ± 438 cm/s, maximum IMT was 1.91 ± 1.17 mm, plaque score was 5.12 ± 6.26, HbA1c level was 7.36 ± 1.52% (57.0 ± 16.6 mmol/mol), LDL-C level was 106.1 ± 28.2 mg/dl, HDL-C level was 60.8 ± 17.6 mg/dl, uric acid level was 5.32 ± 1.35 mg/dl, and urinary albumin level was 157 ± 400 mg/g · Cr). The plaque score was computed by summing the maximum thickness of the intima-media complex measured in millimeters on the near and far walls at each of four divisions of both sides of the carotid arteries on the B-mode image [16].

**Table 1:** Patient characteristics

	**Male**		**Female**		**Total**	
	**Mean ± SD**	**Patients**	**Mean ± SD**	**Patients**	**Mean ± SD**	**Patients**
Age, years	67.9 ± 10.2	196	69.0 ± 10.2	141	68.3 ± 10.2	337
BMI, kg/m^2^	24.4 ± 3.9	196	23.8 ± 4.3	141	24.1 ± 4.1	337
Duration, years	14.1 ± 9.7	196	14.3 ± 9.6	141	14.2 ± 9.7	337
ST-T change or abnormal Q in ECG		Positive in 51		Positive in 27		Positive in 78
Aortic calcification in plain chest radiographs		Positive in 79		Positive in 56		Positive in 135
Minimum value of ABI	1.09 ± 0.14	196	1.08 ± 0.12	141	1.08 ± 0.13	337
Maximum value of PWV	1820 ± 419	196	1840 ± 438	141	1830 ± 438	337
Maximum IMT, mm	2.09 ± 1.14	196	1.66 ± 1.17	141	1.91 ± 1.17	337
Plaque score	5.56 ± 6.47	196	4.51 ± 5.93	141	5.12 ± 6.26	337
HbA1c, % (mmol/mol)	7.25 ± 1.48 (56.0 ± 16.2)	196	7.50 ± 1.58 (58.0 ± 17.3)	141	7.36 ± 1.52 (57.0 ± 16.6)	337
LDL-C, mg/dl	103 ± 26.1	196	111 ± 29.8	141	106.1 ± 28.2	337
HDL-C, mg/dl	55.8 ± 15.8	196	67.9 ± 17.6	141	60.8 ± 17.6	337
uric acid, mg/dl	5.76 ± 1.33	196	4.70 ± 1.11	141	5.32 ± 1.35	337
urinary albumin, mg/g·Cr	173 ± 406	196	135 ± 392	141	157 ± 400	337

Minimum value of ABI (right or left ankle–brachial index), maximum value of PWV (right or left pulse wave velocity).

The plaque score was computed by summing the maximum thickness of the intima-media complex measured in millimeters on the near and far walls at each of four divisions of both sides of the carotid arteries on the B-mode image.

As objective variables are nominally scaled, multivariate logistic analysis was performed with a two-sided test (P < 0.05). Statistical analyses were performed with SPSS ver. 20 and SPSS regression ver. 20 (SPSS Inc., Chicago, IL, USA), except for multicollinearity analysis, which was performed using EZR (Saitama Medical Center, Jichi Medical University), a graphical user interface for R software (The R Foundation for Statistical Computing) [17].

We used an arbitrary number of ischemic artery stenosis as the independent variable. The presence or absence of an ischemic heart disease was represented as the number “1” or “0”, respectively. The dependent variables of gender, ECG findings, and plain chest radiograph findings were also marked in the following way: gender, male (0), female (1); ST-T change or abnormal Q wave on ECG, positive (1), negative (0); and aortic calcification, positive (1), negative (0).

There were 14 independent variables; therefore, we first surveyed for a significant relationship between ischemic heart disease and other factors. Univariate logistic regression analysis was performed between other independent variables and ischemic heart disease, which was defined as a dependent variable.

The unilogistic regression analysis revealed a significant correlation between ischemic heart disease and ECG changes [odds ratio (OR), 7.32; P < 0.01], minimum ABI (OR, 0.0478; P = 0.004), maximum PWV (OR, 1.00; P = 0.01), maximum IMT (OR, 1.50; P = 0.003), plaque score (OR, 1.07; P = 0.004), LDL-C (OR, 0.963; P < 0.001), and UA (OR, 1.47; P = 0.003).

The OR for maximum PWV was 1.00 among significant dependent variables. We omitted this data in subsequent multivariate regression analysis. The OR for LDL-C was 0.960, but this variable did not have a positive relationship with ischemic heart disease. This independent factor was an average value for 5 years. The observation that patients were strictly controlled with statins after an ischemic heart disease or diagnosis of severe atherosclerosis to prevent ischemic heart disease induced a paradoxical result. The higher was the LDL-C, the lower was the ischemic risk. Therefore, we studied the biased population of the statin-untreated and statin-treated groups.

However, many patients with lifestyle diseases, including diabetes, are treated with statins. It is useful to include LDL-C data because most outpatients are in the same situation. We analyzed UA findings in a similar manner.

We evaluated statistical multicollinearity. A strong correlation (correlation coefficient, 0.523; P = 0.00) was observed between maximum IMT and plaque score. The correlated data resulted in multicollinearity in multilogistic regression analysis. We discarded the plaque score and adopted maximum IMT because it had a stronger correlation with ischemic heart disease.

After conducting unilogistic regression analysis between ischemic heart disease and each dependent variable, multilogistic regression analysis was conducted after controlling simultaneously for potential cofounders such as ECG findings, aortic calcification, minimum ABI, maximum IMT, LDL-C, and UA (Table 2). Our results did not have a multicollinearity problem.

**Table 2:** Results of multivariate logistic regression analysis with ischemic heart disease and each independent variable

	**Partial regression coefficient**	**Odds ratio**	**Lower 95% CI**	**Upper 95% CI**	**P-value**
Minimum value of ABI	−1.42	0.242	0.023	2.50	0.23
Aortic calcification in plain chest radiographs	1.55	4.71	1.93	11.5	0.001
ST-T change or abnormal Q in ECG	2.02	7.53	3.17	17.9	<0.001
LDL-C	−0.041	0.960	0.943	0.970	<0.001
Maximum IMT	0.147	1.16	0.803	1.67	0.43
Uric acid	0.211	1.24	0.900	1.70	0.19
Intercept	0.147	1.16			0.94

The chi-square value for the omnibus test of model coefficients was 78.29 (P = 0.00). The chi-square value was 5.669 for the Lemeshow and Hosmer test (P = 0.684). It was denied that the regression equation does not fit.

The regression equation (Y) was as follows:

$$\begin{array}{l} Y=2.02\times \left(\mathrm{ECG}\;\mathrm{change}\right)+1.55\times \left(\mathrm{aortic}\;\mathrm{calcification}\right)\\ \;-1.42\times \left(\min\mathrm{ABI}\right)+0.147\times \left(\max\mathrm{IMT}\right)-0.041\\ \;\times \left(\mathrm{LDL}-C\right)+0.211\times \left(\mathrm{UA}\right)+0.147 \end{array}$$

**Figure 1:**
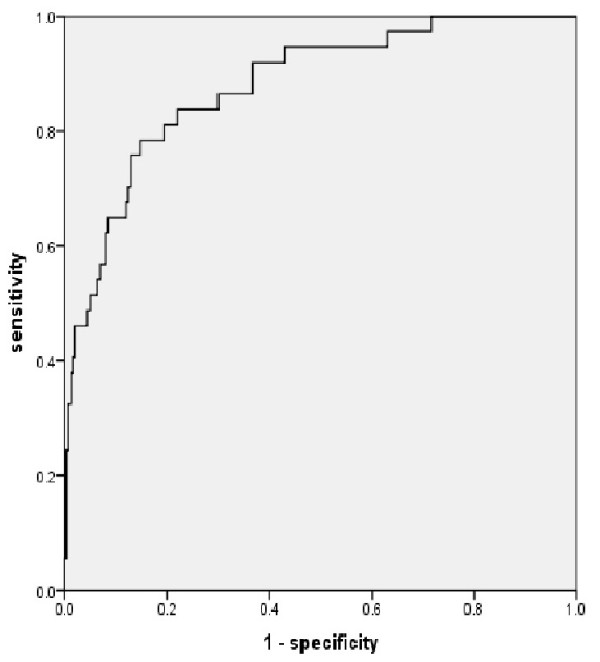
Receiver operating characteristic curves for predicting ischemic heart disease from 2008 to 2013 Area under the curve = 0.879 (0.820–0.938; P = 0.00).

The receiver operating characteristic (ROC) curve for ischemic heart disease in the 337 patients with diabetes derived using the above regression equation is shown in Figure 1. The area under the curve (AUC) was 0.879 (0.820–0.938, P = 0.00; Figure 1).

The logistic equation calculated the cut-off value to be *−*1.84 at the nearest point on the curve from the coordinate (0,1). The classification results for a cut-off value of *−*1.84 are shown in Table 3. According to this cut-off value, 264 patients were in the low-risk category and 73 were in the high-risk category for the 5 years. Eight of the 264 patients in the low-risk group developed ischemic heart disease, while 29 of the 73 patients in the high-risk group suffered ischemic heart disease. Sensitivity was 78.4%, specificity was 85.3%, and the negative predictive value was 97.0%. Therefore, this cut-off value was adequate for screening the disease for diabetic outpatients.

**Table 3:** Onset prediction of ischemic heart disease using the logistic regression equation and the number of patients who actually developed ischemic heart disease

	**Ischemic heart disease (+)**	**Ischemic heart disease (−)**	**Total**
Logistic regression equation (+)	29	44	73
Logistic regression equation (−)	8	256	264
Total	37	300	337

The cut-off value in the logistic regression equation (Y) was calculated from the receiver operating characteristic (ROC) curve. The shortest distance from the coordinates (1-specificity, sensitivity) = (0.0, 1.0) was found on the ROC curve in Figure 1. The value of the logistic regression equation (Y) corresponding to the coordinates was −1.84. The cut-off value for the logistic regression equation (Y) of −1.84 divided the outpatients into low-risk and high-risk groups according to the risk of ischemic heart disease. The outpatients were classified according to the presence or absence of ischemic heart disease.

※ Y = 2.02 × (ECG change) + 1.55 × (aortic calcification) − 1.42 × (min ABI) + 0.147 × (max IMT) − 0.041 × (LDL-C) + 0.211 × (UA) + 0.147.

## Discussions

It is extremely difficult to evaluate ischemic heart disease, which exhibits few symptoms, with simple testing. Generally, tests to evaluate ischemic heart disease include ECG at rest, exercise ECG, electrocardiography, dobutamine stress electrocardiography, a cardiac radioisotope examination, and cardiac computed tomography. ECG at rest is safe and inexpensive, but its specificity and sensitivity is not high, particularly in cases of angina pectoris [18].

Exercise ECG comes second in terms of cost, after ECG at rest. Although an exercise ECG can help in diagnosing structural or functional stenosis, mass screening is difficult because of time constraints. The sensitivity of exercise ECG is 70% and the specificity is 75%; therefore, it does not result in sufficient specificity in a mass screening [19-23]. Moreover, the exercise can trigger a ischemic heart disease, and handicapped patients cannot complete the test [24].

Cardiac ultrasonography shows akinesis of the cardiac wall in patients with unstable angina and infarction, but abnormal findings appear during the late stage of the clinical course. Other tests, including dobutamine stress ECG, cardiac radioisotope examinations, and cardiac CT are also useful at the late stage of the disease; however, these are invasive, pose a radiation risk, or are expensive. Our study used a screening survey and cardiac expertise to develop atherosclerotic evidence.

Our results showed a larger AUC for the ROC curve compared with that in previous studies. Staging of atherosclerosis as it progresses is not uniform organ by organ. A previous study combined the Framingham score and ABI results or those of cervical artery ultrasonography to show the progression of atherosclerosis in a local area. In contrast, our study unites broad information from every organ, including the progression of lower leg atherosclerosis diagnosed by ABI, cervical artery atherosclerosis diagnosed by ultrasonography, and aortic atherosclerosis diagnosed by aortic calcification on plain chest radiographs.

An evaluation of the general progression of atherosclerosis was accomplished with more accurate scoring and a larger AUC. We cannot predict ischemic heart disease in a patient with diabetes using high sensitive C-reactive protein level, HbA1c level, UA findings, or LDL-C level. These patients have been at risk of atherosclerosis for some time. An evaluation of atherosclerotic risk using blood chemistry is useful for predicting long-term future attacks.

A question that deserves further elucidation is the potential use of biomarkers of endothelial cell activation that have been found to be useful predictors of cardiovascular events in chronic diseases associated with increased risk of cardiovascular death. It is the case of angiopietin-2 or osteoprotegerin that have been found to be good predictors of cardiovascular disease and subclinical atherosclerosis in patients with rheumatoid arthritis [25,26].

However, physical and imaging studies, such as electrocardiography, plain chest radiography, and cervical artery ultrasonography are superior for predicting short-term future risk. Evaluation of the real-time progression of atherosclerosis is important for a better predictive result.

There is a possibility that some variations in data from the physical tests or imaging studies were caused by human error. Moreover, the characteristics of an outpatient population are different in each hospital, and our logistic equation could not be directly applied for patients at other hospitals. Nevertheless, the same combination of tests could be used to extract a high-risk group.

In our study, patients with diabetes who visited our hospital for 5 years were retrospectively analyzed. the observation period is long-term and medications such as hypoglycemic agents, statins, and anti-hyperuricemic drugs have been treated for observation period. While some might state that this study was biased compared to the Framingham study, which targeted members of the general population, it is impossible to consider that some diabetes patients already approved for treatment may not undergo treatment intervention. From the perspective of LDL-C level evaluation, the population included both patients being administered statins and patients not being administered statins so this data could have some bias. However, when patients have lifestyle-related diseases such as diabetes, statin administration is usually performed in line with guidelines; in this sense, we believe that it is realistic to evaluate biased data. Accordingly, we decided to also use LDL-C levels as data, regardless of whether patients were being administered. For the same reason, uric acid and blood glucose levels were also adopted regardless of whether patients were being administered antihyperuricemic drugs, hypoglycemic drugs or insulin.

Thus, blood glucose, lipids and blood pressure were managed in diabetes patients according to guidelines and we therefore believe that our prediction model is more practical than other previously reported models for predicting onset of ischemic heart disease in diabetes patients actually undergoing treatment.

Diabetes is a disease that can cause arteriosclerotic disease throughout the body and ischemic heart disease could be more accurately predicted by combining physiological testing to search multilaterally for arteriosclerotic disease without limiting the search to a certain area.

Moreover, in rheumatoid arthritis patients, combining this logistic function and endothelial cell activation markers such as angiopietin-2 and osteoprotegerin could increase ischemic heart disease onset prediction probability.

## Conclusions

We speculate that our screening system is useful and inexpensive compared to the myocardial scintigraphy or cardiac CT inspection. In addition, if we diagnose a high risk patient by using our screening system, we can make an intervention to reduce the risk. Therefore, our screening method is very useful, less invasive, high efficient, and cost-effective for diabetic cardiovascular risk. It is very important to score the risk for a stepped-care approach to prevent the progression of diabetes physically and mentally.

## Competing interests

The authors declare that they have no competing interests.

## Authors’ contributions

TK recruited the patients, researched the data, wrote the manuscript, wrote the protocol, edited and reviewed the manuscript. MSS, RDH, and RAH recruited the patients and researched the data. TO researched the data and contributed to the discussion. HK is the guarantor of this work and, as such, had full access to all the data in the study and takes responsibility for the integrity of the data and the accuracy of the data analysis. All authors read and approved the final manuscript.
